# Prevalence and Determinants of Smartphone and Social Media Addiction Among Medical Residents in Tunisia

**DOI:** 10.1192/j.eurpsy.2026.10825

**Published:** 2026-07-31

**Authors:** N. Rmadi, R. Affes, A. Hrairi, N. Kotti, M. Hajjaji, K. Jmal Hammami

**Affiliations:** 1 Occupational medicine department, Hedi Chaker university hospital; 2 Family medecine department, Faculty of medecine of Sfax, Sfax, Tunisia

## Abstract

**Introduction:**

Digital technologies have profoundly transformed medical practice, yet problematic use of smartphones and social media is increasingly recognized as an addictive disorder. Medical residents, exposed to heavy workloads and psychosocial stressors, may be particularly vulnerable.

**Objectives:**

To estimate the prevalence of smartphone and social media addiction and identify associated sociodemographic, academic, and health-related determinants among Tunisian medical residents.

**Methods:**

A national cross-sectional study was conducted between January and April 2025 among 429 residents from the four medical faculties in Tunisia. Data were collected via an anonymous online questionnaire including the Smartphone Addiction Scale–Short Version (SAS-SV), the Bergen Social Media Addiction Scale (BSMAS), and sociodemographic, academic, and health-related variables. Associations were explored using bivariate analyses and logistic regression models.

**Results:**

Almost half of the residents (49.9%) met the criteria for smartphone addiction, while 25.6% were addicted to social media. Mean daily smartphone use exceeded five hours, with social networking as the leading motivation (59.2%). Female gender (OR=2.1; 95%CI [1.4–3.1]; p<0.001) and psychiatric history (OR=2.5; 95%CI [1.4–4.4]; p=0.001) were significantly associated with digital addiction. Residents in family medicine were more prone to social media addiction (p=0.004).

**Conclusions:**

Digital addiction is highly prevalent among Tunisian medical residents, particularly among women and those with prior psychiatric disorders. These findings highlight the importance of implementing early screening, targeted awareness initiatives, and digital hygiene interventions specifically adapted to the context of residency training programs.

**Disclosure of Interest:**

None Declared

